# Clustering Treatment Outcomes in Women with Gambling Disorder

**DOI:** 10.1007/s10899-021-10092-5

**Published:** 2021-12-21

**Authors:** Milagros Lizbeth Lara-Huallipe, Roser Granero, Fernando Fernández-Aranda, Mónica Gómez-Peña, Laura Moragas, Amparo del Pino-Gutierrez, Eduardo Valenciano-Mendoza, Bernat Mora-Maltas, Isabel Baenas, Mikel Etxandi, José M. Menchón, Susana Jiménez-Murcia

**Affiliations:** 1 Department of Psychiatry, Hospital Universitari de Bellvitge, L′Hospitalet de Llobregat, IDIBELL and CIBERObn. C/ Feixa Llarga S/N, Hospitalet de Llobregat, 08907 Barcelona, Spain; 2 Department of Psychobiology and Methodology, Universitat Autònoma de Barcelona - UAB, Barcelona, Spain; 3 Ciber Fisiopatología Obesidad Y Nutrición (CIBERobn), Instituto Salud Carlos III, Madrid, Spain; 4 Psychiatry and Mental Health Group, Neuroscience Program, Institut d′Investigació Biomèdica de Bellvitge - IDIBELL, L′Hospitalet de Llobregat, Barcelona, Spain; 5 Department of Clinical Sciences, School of Medicine and Health Sciences, Universitat de Barcelona–UB, L′Hospitalet de Llobregat, Barcelona, Spain; 6 Department of Public Health, Mental Health and Perinatal Nursing, School of Nursing, Universitat de Barcelona–UB, Barcelona, Spain; 7 CIBER Salud Mental (CIBERSam), Instituto Salud Carlos III, Madrid, Spain

**Keywords:** Clustering, Cognitive behavioral therapy, Gambling disorder, Survival analysis, Women

## Abstract

The rising prevalence of gambling disorder (GD) among women has awakened considerable interest in the study of therapeutic outcomes in females. This study aimed to explore profiles of women seeking treatment for GD based on a set of indicators including sociodemographic features, personality traits, clinical state at baseline, and cognitive behavioral therapy (CBT) outcomes. Two-step clustering, an agglomerative hierarchical classification system, was applied to a sample of *n* = 163 women of ages ranging from 20 to 73 years-old, consecutively attended to by a clinical unit specialized in the treatment of G. Three mutually exclusive clusters were identified. Cluster C1 (*n* = 67, 41.1%) included the highest proportion of married, occupationally active patients within the highest social status index. This cluster was characterized by medium GD severity levels, the best psychopathological functioning, and the highest mean in the self-directedness trait. C1 registered 0% dropouts and only 14.9% relapse. Cluster C2 (*n *= 63; 38.7%) was characterized by the lowest GD severity, medium scores for psychopathological measures and a high risk of dropout during CBT. Cluster C3 (*n* = 33; 20.2%) registered the highest GD severity, the worst psychopathological state, the lowest self-directedness level and the highest harm-avoidance level, as well as the highest risk of relapse. These results provide new evidence regarding the heterogeneity of women diagnosed with GD and treated with CBT, based on the profile at pre- and post-treatment. Person-centered treatments should include specific strategies aimed at increasing self-esteem, emotional regulation capacities and self-control of GD women.

**Supplementary Information:**

The online version contains supplementary material available at 10.1007/s10899-021-10092-5.

## Introduction

The onset and progression of gambling disorder (GD) is characterized by clinically significant impairment in multiple areas of functioning, including psychological, working, social and even financial (Langham et al., 2016; Shannon et al., 2017). Epidemiological studies estimate the prevalence of GD among the general population in developed countries at around 1%, and the prevalence of problematic gambling behavior at around 7% (Calado and Griffiths, 2016). These rates give idea of the potential harms and social costs associated with GD, and highlight the urgent need on strength evidence-based intervention (and prevention) plans. These treatments should deliver precise strategies to patients based on the patients’ individual variability.

Sex seems to have been considered a relevant risk factor for the onset and evolution of disordered gambling, and previous studies have obtained ratios of around 1/4 for frequency among females/males (Karlsson and Håkansson, 2018). However, in recent decades, the incidence of GD has risen in all sectors of the population, including women (Gainsbury et al., 2015; Håkansson and Ford, 2019). It is also known that individuals with gambling problems are often not properly diagnosed and/or untreated, even in clinical settings, which may have led to underestimation of the real prevalence of GD (Blanco et al., 2015; Quintero, 2016; Rash et al., 2016). This situation is aggravated among women, who are more reluctant to seek therapeutic help despite the severe negative consequences of GD: some women prioritize treatment for different comorbid conditions over GD (such as depression or anxiety), other women may conceal the symptoms due to the social stigma attached, and other simply accept their addiction as a lifestyle (Babić et al., 2018; Bischof et al., 2014; Braun et al., 2014; Coriale et al., 2015).

Some studies have observed that at the beginning of treatment GD severity is similar among men and women (number of gambling symptoms, level of urgency related to the gambling behavior or cognitive biases associated with gambling expectations) (Grant, Chamberlain, et al., 2012; Grant, Odlaug, et al., 2012; G. Mestre-Bach et al., 2016a, b; Smith et al., 2015). However, remarkably different results have been obtained in other studies, which suggest the existence of distinct profiles in men and women regarding gambling severity (Susana Jiménez-Murcia et al., 2020; Ronzitti, Lutri, et al., 2016; Ronzitti, Soldini, et al., 2016), as well as in other compulsive-related neurocognitive domains (Mallorquí-Bagué et al., 2021), and in the role of the urge to gamble and gambling-related cognitions in the tracking correlations of GD over time (Dunsmuir et al., 2018). Empirical evidence has also showed that patients’ sex could modulate the relationships between the multiple variables that can be used to explain gambling severity and the development of the disorder. For example, comorbidity patterns have revealed that GD women report higher levels of depression and anxiety, while GD in men increases the likelihood of substance-related disorders (alcohol and drugs) (Di Nicola et al., 2015; Dion et al., 2015; Dowling et al., 2015; Pilver et al., 2013a,2013b; Ronzitti et al., 2016; Ronzitti, Soldini, et al., 2016; Sundqvist and Rosendahl, 2019; Tackett et al., 2017). It has also been claimed that the association between GD and other comorbid psychiatric conditions is stronger among women than among men (Håkansson et al., 2018; Hartmann and Blaszczynski, 2018), and that previous mental illness is also a stronger risk factor for later onset of problematic gambling among women (Haw and Holdsworth, 2016; Sundqvist and Rosendahl, 2019). The age of onset of GD and its course also evidenced a sex-related profile, characterized by the well-known telescopic effect: women tend to commence gambling activity later in life but progress to gambling-related problems more quickly compared to men (Grant et al., 2012; Grant, Odlaug, et al., 2012; Slutske et al., 2015). Gambling preferences also seem to be strongly related with sex (Hing et al., 2016a,2016b): women tend to choose games classified as non-strategic (characterized by the subject's inability to exercise any type of control over the outcome of the bet: lotteries, bingo and slot machines, while men present a higher prevalence for strategic games (characterized by gamblers being able to use their knowledge to predict outcomes, such as poker, craps/dice games or sports betting). Finally, a number of other systematic differences have also been reported between the GD pathogenesis of men and women (Fattore et al., 2014; S. Jiménez-Murcia et al., 2014; Smith et al., 2015). For example, it has been claimed that women often use gambling activity mainly as a maladaptive coping strategy to avoid negative mood states, while many other gambling motives have been described as stronger among men [enhancement (playing for pleasure and for increasing positive emotions), avoiding boredom, socializing, excitement, competing with others or even financial reasons (making money through gambling activities)] (Grant, et al., 2012a, 2012b; Hing et al., 2016a,2016b; Mathieu et al., 2020; Moragas et al., 2015). And regarding personality traits, women with GD tend to have lower mean scores for impulsivity and sensation seeking than men (Hodgins and Holub, 2015).

Women seeking care for gambling-related problems usually follow the same treatment protocols as men, which is often cognitive-behavioral therapy, CBT (An et al., 2017). This evidence-based therapy is currently considered the gold standard for GD treatment (Abbott, 2019; Challet-Bouju, Bruneau, IGNACE Group, Victorri-Vigneau, and Grall-Bronnec, 2017; Petry et al., 2017), and its effectiveness in the short and medium terms has been widely demonstrated (Merkouris et al., 2016). And although a large number of studies have provided evidence of the effectiveness of CBT for GD (Cowlishaw et al., 2012; Merkouris et al., 2016), most research has analyzed data collected from men (Tolchard, 2017). The few studies found in the literature within clinical samples with women suggest that CBT could be more effective among male sex (in the short term and at 6 months of follow up) (Toneatto and Wang, 2009).

On the other hand, most studies exploring CBT outcomes within GD were carried out with a variable-centered methodology (the dominant approach in the field of psychopathological research), characterized by analyzing the relationships and covariances between a set of variables through classical procedures such as generalized linear models (Von Eye and Bogat, 2006). Few studies have used person-centered alternative approaches like latent classes and other classification methods, based on analysis of the specific individuals’ processes, their functioning, and the behavioral expression of the different domains (Howard and Hoffman, 2017). And very few works have combined person-centered and variable-centered methods as complement analytical techniques (Laursen and Hoff, 2006; Muthén and Muthén, 2000): at the first step, person-centered methods identify subgroups of people who share specific profiles, and in the second step variable-centered methods operate on a higher level of generality with the aim of revealing the connection between features.

### Objectives

The aim of this study was to explore the existence of empirical clusters in a sample of women diagnosed with GD and treated with CBT, based on a large set of indicator/predictor variables including sociodemographics, personality traits, clinical state at baseline (prior to treatment), and therapy outcomes (mainly the risk of dropout and relapse). Based on the cumulate evidence reported in the scientific literature for men diagnosed with GD, we hypothesized that this disorder could be conceptualized as a mixed group with differentiated latent subgroups among women. These underlying latent clusters would represent distinct GD profiles. The lack of previous research into CBT outcomes in GD women based on person-centered approaches meant we were unable to make a priori assumptions about the expected number of clusters.

## Methods

### Participants

The sample used in this study included *n* = 163 women being attended to consecutively at the Pathological Gambling and other Behavioral Addictions Unit at the Bellvitge University Hospital (Barcelona). Inclusion criteria were being female, adult age (18 and above) and meeting clinical criteria for GD (according to the DSM-5 taxonomy).

Chronological age was in the range of 20 to 73 years (mean = 47.8, SD = 11.3). Most of the participants had low education levels (primary or less, 55.2%), were single (39.3%) or married (38.0%), were employed (55.2%) and belonged to low socioeconomic levels (62.6%). The mean onset age was 36.7 yrs-old (SD = 11.5) and the mean duration of the gambling habit was 5.7 years (SD = 5.7). The most prevalent gambling preference was non-strategic (85.9%) (strategic gambling was reported by 4.9% of the participants and mixed gambling by 9.2%).

### Measures

*Diagnostic Questionnaire for Pathological Gambling* (according to DSM criteria) (Stinchfield, 2003). This self-report questionnaire was initially developed to diagnose the presence of GD using 19 items based on DSM criteria. Both GD diagnoses are available, for the DSM-IV-TR (American Psychiatric Association, 2010) and the DSM-5 versions (American Psychiatric Association, 2013). The Spanish psychometrical adaptation of this tool achieved adequate properties (Cronbach’s alpha α = 0.81 for a population-based sample and α = 0.77 for a clinical sample) (Susana Jiménez-Murcia et al., 2009). The internal consistency achieved in this study was adequate (α = 0.77).

*Symptom Checklist-Revised* (SCL-90-R) (Derogatis, 1997). This self-report questionnaire is used to assess psychological state by means of 90 items structured into nine primary (first order) dimensions (somatization, obsessive–compulsive, interpersonal sensitivity, depression, anxiety, hostility, phobic anxiety, paranoid ideation, and psychoticism), and three global indices [global severity index (GSI), total positive symptoms (PST), and positive symptoms discomfort index (PSDI)]. The Spanish psychometrical adaptation of this tool obtained adequate properties (mean Cronbach’s alpha α = 0.75) (Gonzalez De Rivera et al., 1989). The internal consistency in our sample was also in the adequate to excellent range (from α = 0.77 for paranoid ideation scale to α = 0.983 for the global indices).

*Temperament and Character Inventory-Revised* (TCI-R) (Cloninger, Przybeck, Syrakic, and Wetzel, 1994). This self-report questionnaire was developed to assess personality traits by means of 240 items based on Cloninger's multidimensional model. The tool is structured into 7 factors [4 for temperament (novelty seeking, harm avoidance, reward dependence, and persistence), and 3 for character (self-directedness, cooperation, and self-transcendence)]. The Spanish psychometrical adaptation of the tool obtained adequate properties (mean Cronbach’s alpha α = 0.87) (Gutiérrez-Zotes et al., 2004). The internal consistency in the study sample was in the adequate to good range (from α = 0.708 for novelty seeking to α = 0.840 for self-transcendence).

*Other variables.* Other additional data assessed using a semi-structured interview were also analyzed. This tool covered socio-demographic characteristics (sex, marital status, education level and employment status), as well as the socio-economic status index according to Hollingshead’s scale (based on employment status, participants’ level of education and occupational prestige) (Hollingshead, 2011). Patients were also asked about gambling-related variables: the onset age and the duration of the GD, bets per gambling episode, and cumulated debts due to the gambling addiction.

### CBT Program

CBT was implemented in this study as a time-limited technique across 16 weekly individual sessions lasting 90 min each. The main objective was to achieve full abstinence from all types of gambling. To achieve this purpose, different strategies were implemented to regulate the patients’ negative emotions, to reduce arousal levels in the presence of any stimuli that trigger the urge to gamble, and to increase self-control of gambling. Throughout the process, women received feedback regarding the improvement of their self-efficacy and all efforts made to achieve recovery are reinforced.

All the CBT sessions were structured within an outpatient program in the Hospital Unit. The complete program was presented and developed by a qualified CBT, a clinical expert on problematic and disordered gambling. The program included a first step consisting of a psycho-education session, focused on the following topics: (a) providing knowledge about the endo-phenotypes of GD, the onset and development of the disorder, and vulnerability and protective factors related to its course (onset and progression); (b) explaining the rationale behind CBT; (c) teaching methods to identify the dysfunctional thoughts and feelings related to gambling activity; (d) explaining cognitive restructuring techniques and problem-solving techniques addressed at generating alternative functional feelings and thoughts of wellbeing (including relaxation procedures); and (e) providing knowledge regarding stimulus control procedures [such as avoidance of potential triggers, financial planning and self-exclusion (from both land-based and online gambling)].

During the initial phase of the CBT program, participants also received column sheets that they were instructed to fill in each day, detailing situations where they felt unwell (irritable or anxious), behaviors related to gambling, automatic thoughts, an objective examination of those automatic thoughts (including counterevidence), adaptive thoughts, and changes in feeling and behavior.

During the 16 treatment sessions, patients applied CBT techniques, completed the column sheets and attended individual sessions at which questions and opinions regarding the progression of the therapy were exchanged. Changes in gambling behavior and overall psychological state, as well as the learned skills, were evaluated at the end of the treatment.

Different studies have shown the short- and long-term effectiveness of the CBT used in this study in GD samples (S. Jiménez-Murcia et al., 2007, 2015, 2019; Susana Jiménez-Murcia et al., 2016; Jiménez-Murcia, Tremblay, et al., 2017; Jiménez-Murcia, Fernández-Aranda, et al., 2017).

### Procedure

The study was carried out in accordance with the Declaration of Helsinki principles, and was approved by the Ethics Committee of the University Hospital of Bellvitge (Ref: PR329/19). All patients provided signed informed consent to participate in the research. There was no financial or other compensation for being part of the study sample.

The variables recruited at baseline (prior to the treatment) were analyzed in an assessment process consisting of a single session lasting about 90 min. Data for the semi-structured interview were collected by psychologists and psychiatrists with extensive experience of the treatment of behavioral addictions. The clinicians also helped the participants to complete the self-report questionnaires in order to guarantee that no data were missing (for example, due to lack of understanding).

### Statistical Analysis

SPSS24 for Windows (IBM-Corp, 2016) was used for the statistical analysis. First, two-step clustering was used to identify the latent empirical classes. This clustering technique is an agglomerative hierarchical analysis used to explore the existence of natural groupings, useful to handle categorical and quantitative variables, and with the advantage of automatically selecting the optimal number of clusters. The log-likelihood distance was used in this study, and the best model was determined using the Schwarz Bayesian Information Criterion (BIC) and Akaike’s Information Criterion (AIC). This method considers the optimal number of latent classes in the model with the largest ratio of changes for the BIC and AIC, as well as the largest ratio of distances measured when comparing the current number of clusters against the previous number. Two-step clustering uses the Silhouette index (a cohesion-separation measure to show how similar individuals are to their own cluster compared to other clusters) to assess the quality of the clustering (Rousseeuw, 1987). The Silhouette coefficient is in the range of − 1 to + 1, with high values indicating adequate matching in one’s own cluster and poor matching in other clusters (values lower than 0.30 are considered poor fits, between 0.30 and 0.50 are fair, and higher than 0.50 are good). The indicator/predictor variables used for two-step clustering in this study were personality traits (measured at baseline using the TCI-R), global psychopathological distress (measured at baseline with the SCL-90R GSI), GD severity (number of DSM-5 criteria for gambling, debts due to the GD), age, onset age, and relapse and dropout outcomes of CBT. The final model selected for this study was based on the following criteria (Nylund et al., 2007): (a) adequate goodness-of-fit (based on a Silhouette cohesion and separation index); (b) adequate clinical interpretability; and (c) number of individuals in each group to guarantee statistical power.

Second, the comparison between the latent empirical clusters obtained in this study was based on chi-square tests (χ^2^) for categorical variables and on analysis of variance (ANOVA) procedures for quantitative measures. The estimation of the effect sizes for the proportion and mean differences was based on the standardized Cohen’s-*d* coefficient, considering poor-low effect size for |*d*|> 0.20, moderate-medium for |*d*|> 0.5, and large-high for |*d*|> 0.80 (Kelley and Preacher, 2012). In addition, the increase in Type-I errors due to the multiple statistical tests for comparing clusters was controlled with the Finner method (included in the stepwise familywise error rate procedures) (Finner, 1993).

Third, survival analysis was used to describe the hazard rate of dropout and relapse and the comparison between the empirical clusters. This technique is used for modeling censored data, which occurs in longitudinal studies when patients withdraw from the study (that is, manage to ‘survive’ to the end of the follow-up, or are lost to the follow-up without event occurrence at last measurement time) (Aalen et al., 2008; Singer and Willett, 2003). This study used the Kaplan–Meier (product-limit) estimator to describe the probability of women “living” to the end of the CBT (in the study, survival is considered to be the time without dropout or without the presence of relapse episodes). Comparison between the groups for the survival function was based on the Log Rank (Mantel-Cox) test.

## Results

### Clustering Procedure

The results for the two-step clustering are displayed in Table S1 (supplementary material), for models ranging from 1 to 8 clusters. The optimal number of latent clusters automatically selected by the system was the 3-cluster model: it achieved the highest ratio distance (2.49) and the highest Silhouette index (0.30, in the fair range). This 3-cluster solution was also selected as the final model since it also achieved good clinical interpretation, and discriminative capacity to differentiate between features related to the sociodemographics, the clinical profile at baseline and the CBT outcomes.

**Fig. 1:**
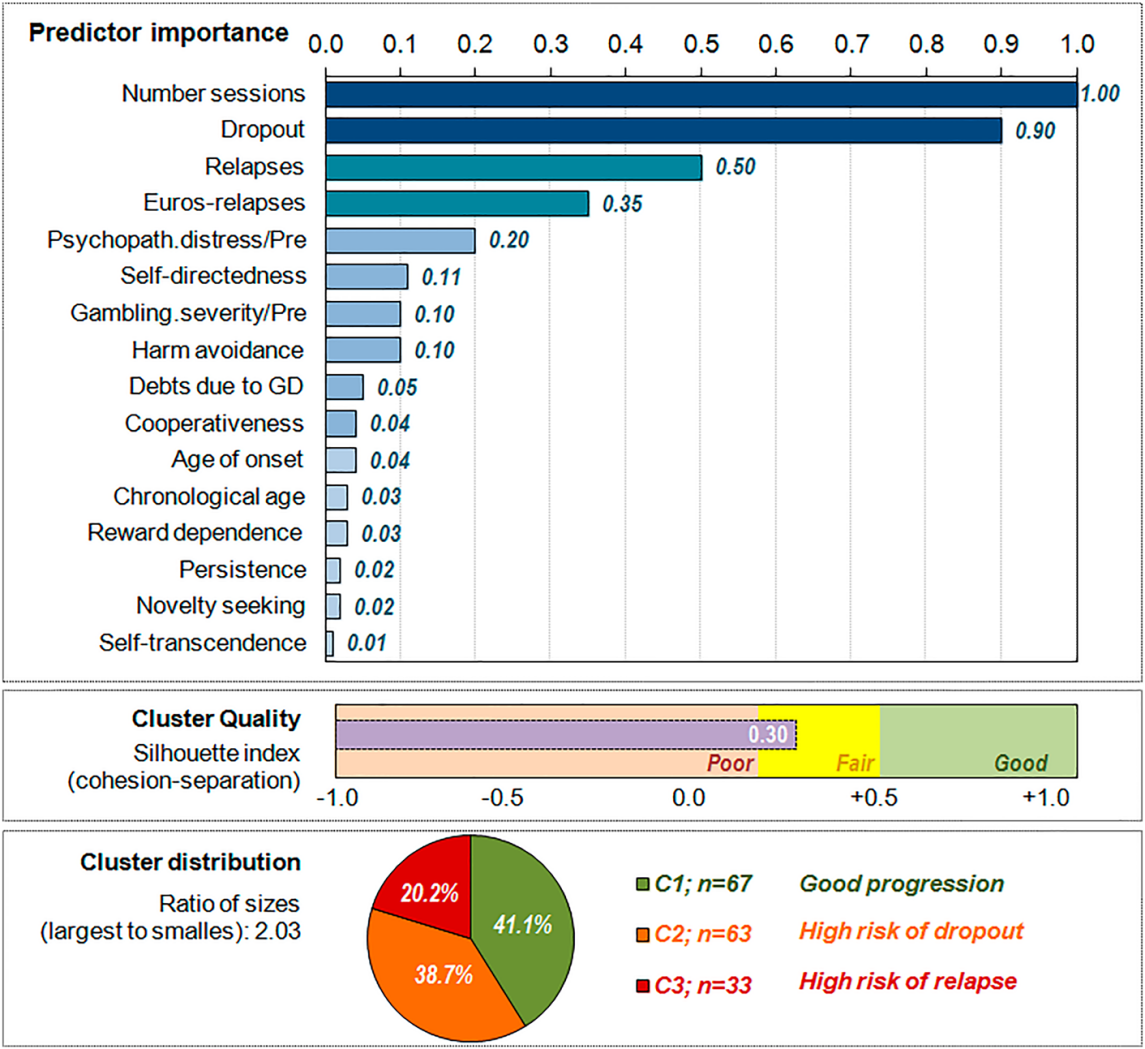
Results of the clustering procedure (*n* = 163)

The ordered bar-chart in Fig. 1 shows the relative importance of each indicator/predictor variable in the clustering. This measure is in the range of 0 to 1, and provides an estimation of the discriminative capacity of the indicators: the greater the relevance, the less likely it is for changes between clusters for the variable to be attributable to chance. The variable with the highest relevance in the clustering in the study was the number of sessions attended during the CBT, closely followed by the presence of dropout. On the contrary, the variables with the lowest relevance were personality trait dimensions (self-transcendence, novelty-seeking and persistence). Figure 1 also contains a graphic representation of the Silhouette index in the study, and the frequency distribution for the clusters (ratio size between the largest and lowest clusters was 2.03).

### Comparison Between Clusters

The upper part of Table 1 contains a comparison between the 3 clusters for the sociodemographic variables analyzed in this study. According to the variables that achieved significant result and/or effect size in the mild-moderate to large-high range, cluster 1 was characterized by including the higher proportion of married, occupationally active patients with the highest social status index. On the contrary, clusters 2 and 3 were characterized by including the higher proportion of non-married and unemployed women with the lowest social status indexes. No differences between clusters 2 and 3 were found in terms of sociodemographics.

**Table 1:** Comparison between the clusters for the sociodemographic and clinical variables at baseline

	Cluster-1		Cluster-2		Cluster-3		Cluster-1 vs		Cluster-1 vs		Cluster-2 vs	
	*n* = 67		*n* = 63		*n* = 33		Cluster-2		Cluster-3		Cluster-3	
	*n*	*%*	*n*	*%*	*n*	*%*	*p*	*|d|*	*p*	*|d|*	*p*	*|d|*
Education												
Primary or less	32	47.8%	37	58.7%	21	63.6%	.380	0.22	.327	0.32	.819	0.10
Secondary	29	43.3%	23	36.5%	10	30.3%		0.14		0.27		0.13
University	6	9.0%	3	4.8%	2	6.1%		0.17		0.11		0.06
Marital status												
Single	24	35.8%	25	39.7%	15	45.5%	.492	0.08	.083	0.20	.465	0.12
Married-couple	31	46.3%	23	36.5%	8	24.2%		0.20		**0.51**^**†**^		0.27
Divorced-Separated	12	17.9%	15	23.8%	10	30.3%		0.15		0.29		0.15
Employment												
Unemploy	22	32.8%	32	50.8%	19	57.6%	.038*	0.37	.018*	**0.52**^**†**^	.527	0.14
Employed	45	67.2%	31	49.2%	14	42.4%		0.37		**0.52**^**†**^		0.14
Social Mean-high / High	6	9.0%	2	3.2%	1	3.0%	.023*	0.25	.650	0.26	.762	0.01
Mean	13	19.4%	5	7.9%	5	15.2%		0.34		0.11		0.23
Mean-low	11	16.4%	12	19.0%	6	18.2%		0.07		0.05		0.02
Low	37	55.2%	44	69.8%	21	63.6%		0.30		0.17		0.13
	*Mean*	*SD*	*Mean*	*SD*	*Mean*	*SD*	*p*	*|d|*	*p*	*|d|*	*p*	*|d|*
Chronological age (yrs-old)	48.7	11.8	47.9	11.9	45.8	9.2	.656	0.08	.227	0.28	.405	0.19
Age of onset GD (yrs-old)	36.6	11.4	38.1	12.2	34.2	10.3	.470	0.12	.333	0.22	.123	0.34
Duration GD (yrs)	6.0	6.2	5.2	4.8	6.0	6.3	.448	0.14	.980	0.00	.552	0.13
Number of DSM-5 criteria	7.1	1.7	6.3	2.1	7.8	1.1	.023*	0.38	.045*	**0.54**^**†**^	.001*	**0.90**^**†**^
Bets (mean-episode, euros)	78	112	65	122	102	130	.534	0.11	.341	0.20	.148	0.30
Bets (max-episode, euros)	488	752	421	680	746	1191	.647	0.09	.148	0.26	.072	0.34
Debts due to GD (euros)	5844	7690	3098	6191	1545	3851	.017*	0.39	.002*	**0.71**^**†**^	.268	0.30
SCL-90R: Somatization	1.24	0.78	1.57	0.78	2.13	0.77	.017*	0.42	.001*	**1.15**^**†**^	.001*	**0.72**^**†**^
SCL-90R: Obsessive/comp	1.40	0.93	1.69	1.04	2.12	1.03	.107	0.29	.001*	**0.73**^**†**^	.043*	0.42
SCL-90R: Interper.sensitivity	1.36	0.87	1.54	0.96	2.23	0.72	.265	0.19	.001*	**1.08**^**†**^	.001*	**0.82**^**†**^
SCL-90R: Depressive	1.10	0.88	1.42	0.90	2.31	0.88	.043*	0.36	.001*	**1.37**^**†**^	.001*	**1.00**^**†**^
SCL-90R: Anxiety	1.75	0.94	2.27	1.00	2.69	0.89	.002*	**0.53**^**†**^	.001*	**1.03**^**†**^	.040*	0.45
SCL-90R: Hostility	1.24	0.93	1.60	1.02	2.15	1.00	.034*	0.38	.001*	**0.95**^**†**^	.010*	**0.54**^**†**^
SCL-90R: Phobic anxiety	0.88	0.82	1.12	0.92	1.68	0.92	.113	0.28	.001*	**0.92**^**†**^	.004*	**0.61**^**†**^
SCL-90R: Paranoid Ideation	0.66	0.85	0.93	0.97	1.58	1.11	.108	0.30	.001*	**0.93**^**†**^	.002*	**0.62**^**†**^
SCL-90R: Psychotic	0.95	0.77	1.30	0.85	1.87	0.94	.017*	0.44	.001*	**1.07**^**†**^	.002*	**0.63**^**†**^
SCL-90R: GSI score	0.94	0.77	1.19	0.82	1.80	0.83	.081	0.31	.001*	**1.07**^**†**^	.001*	**0.74**^**†**^
SCL-90R: PST score	50.0	21.4	56.7	19.4	70.0	16.1	.052	0.33	.001*	**1.06**^**†**^	.002*	**0.75**^**†**^
SCL-90R: PSDI score	2.07	0.64	2.34	0.63	2.66	0.63	.017*	0.42	.001*	**0.92**^**†**^	.021*	**0.50**^**†**^
TCI-R: Novelty seeking	109.7	11.5	109.2	15.2	110.6	16.6	.827	0.04	.779	0.06	.648	0.09
TCI-R: Harm avoidance	107.2	16.1	109.3	17.0	120.5	20.7	.487	0.13	.001*	**0.71**^**†**^	.003*	**0.59**^**†**^
TCI-R: Reward dependence	102.2	16.4	101.1	13.4	99.5	14.1	.656	0.08	.397	0.18	.634	0.11
TCI-R: Persistence	104.6	16.7	104.5	21.8	102.7	22.3	.969	0.01	.652	0.10	.679	0.08
TCI-R: Self-directedness	124.7	19.9	116.0	21.4	106.7	17.6	.015*	0.42	.001*	**0.96**^**†**^	.032*	**0.51**^**†**^
TCI-R: Cooperativeness	135.2	15.1	132.4	14.8	129.6	16.4	.291	0.19	.085	0.36	.397	0.18
TCI-R: Self-Transcendence	68.0	14.9	68.9	19.3	69.7	19.3	.790	0.05	.663	0.09	.831	0.04

Cronbach’s-alpha in the sample. SD: standard deviation. *Bold: significant comparison

^†^Bold: effect size into the mild-moderate (|*d*|> 0.50) to large-high range (|*d*|> 0.80)

The lower part of Table 1 contains a comparison between the 3 clusters for the clinical measures recorded at baseline (prior the CBT). Cluster 1 was characterized as reporting medium GD severity (according to the number of DSM-5 criteria), the highest mean of debts related to the gambling activity, the best psychopathological state (lowest mean scores in the SCL-90R scales) and the highest mean in the self-directedness trait. Cluster 3, however, registered the highest GD severity (according to the number of DSM-5 criteria), the worst psychopathological state, the lowest self-directedness level and the highest harm-avoidance level. Cluster 2 was characterized by the lowest GD severity (based on the number of DSM-5 criteria), medium scores in the psychopathological scales (compared to both cluster 1 and 3), a harm-avoidance level similar to cluster 1 and lower than cluster 3, and a medium score for self-directedness (compared with both cluster 1 and 2).

The first block in Table 2 contains a comparison between the 3 clusters for the risk of dropout and relapse during CBT treatment. Cluster 1 registered 0% dropouts, and only 14.9% relapses. All patients in cluster 2 drop out from the treatment, and 19% also registered relapse. Cluster 3 was defined by grouping the highest risk of relapse (97% of patients in this subgroup registered gambling-episodes during the treatment) and 21.2% dropouts.

**Table 2:** Comparison between the clusters for the CBT outcomes

	Cluster-1		Cluster-2		Cluster-3		Cluster-1 vs		Cluster-1 vs		Cluster-2 vs	
	*n* = 67		*n* = 63		*n* = 33		Cluster-2		Cluster-3		Cluster-3	
	*n*	*%*	*n*	*%*	*n*	*%*	*p*	*|d|*	*p*	*|d|*	*p*	*|d|*
Dropout	0	0.0%	63	100.0%	7	21.2%	.001*	**3.14**^**†**^	.001*	**0.96**^**†**^	.001*	**2.18**^**†**^
Relapses	10	14.9%	12	19.0%	32	97.0%	.531	0.11	.001*	**2.00**^**†**^	.001*	**1.89**^**†**^
	*Mean*	*SD*	*Mean*	*SD*	*Mean*	*SD*	*p*	*|d|*	*p*	*|d|*	*p*	*|d|*
Number relapses	0.25	0.64	0.27	0.68	3.88	4.34	.964	0.02	.001*	**1.17**^**†**^	.001*	**1.16**^**†**^
Euros relapses	9.0	26.0	33.2	93.3	430.8	576.5	.603	0.35	.001*	**1.03**^**†**^	.001*	**0.96**^**†**^

SD: standard deviation. *Bold: significant comparison

^†^Bold: effect size into the mild-moderate (|*d*|> 0.50) to large-high range (|*d*|> 0.80)

The second block in Table 2 contains the number of relapses registered during the CBT and the euros spent on the gambling activity in the relapse episodes. While no differences emerged when comparing clusters 1 and 2 for these two measures, cluster 3 registered the highest means compared with the other subgroups.

Based on the results shown in Tables 1 and 2, the empirical clusters obtained in this study were labeled: cluster 1 “good progression”, cluster 2 “high risk of dropout” and cluster 3 “high risk of relapse”.

**Fig. 2:**
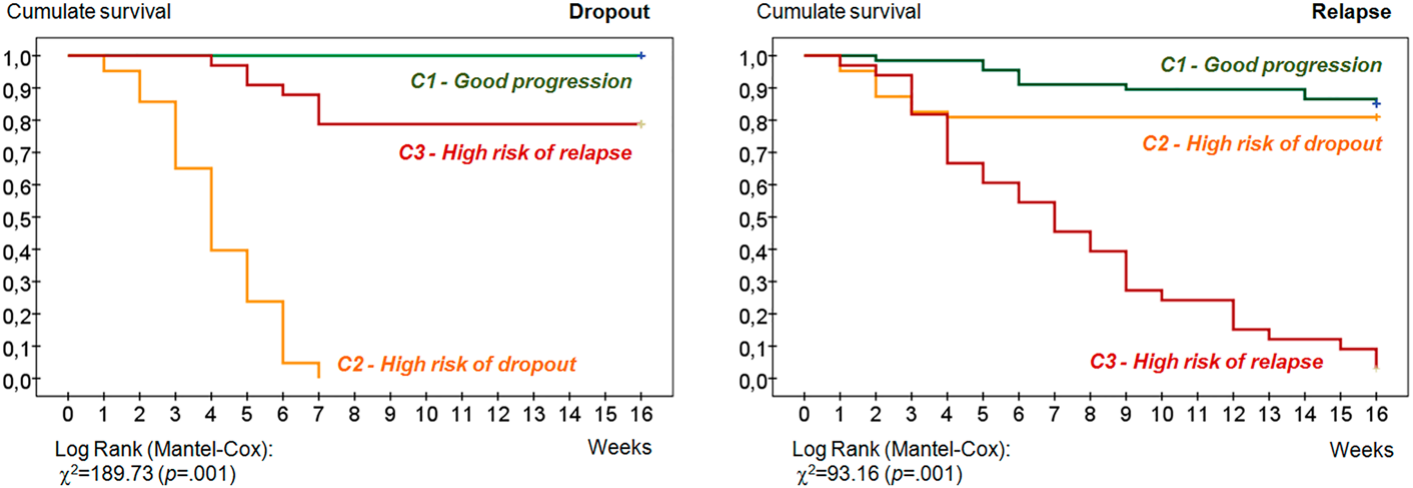
Kaplan–Meier functions for the rate to dropout and relapse (*n* = 163)

Figure 2 contains the Kaplan–Meier functions to the time of dropout and relapse. The cumulate survival to dropout shows that 100% of the patients in cluster 1 did not drop out at any stage of CBT follow-up. The dropouts registered for patients included in clusters 2 and 3 occurred during weeks 1 to 7 (before ending the second month of the treatment). Regarding relapses, the gambling episodes reported by 97% of patients in cluster 3 were registered throughout CBT follow-up. 14.9% of patients with relapses in cluster 1 also reported that these gambling episodes occurred throughout the whole treatment. The relapses registered in cluster 2 were all reported during weeks 1 to 4.

## Discussion

This study aimed to explore clusters of women seeking treatment for GD based on sociodemographic features, clinical state at baseline and CBT outcomes. The 3-cluster solution was selected as optimal: C1 clustered patients with good progression during the treatment, C2 patients with poor progression to dropout, and C3 patients with poor progression to relapse. The identification of these empirical groups evidences the heterogeneity of progression during treatment in women with GD, which can be described in separate profiles based on the demographic and clinical features.

Cluster C1 included the patients with the best progression during treatment (low rates of dropout and relapse), and was characterized by the highest proportion of women that are married, with the highest social status indexes, employed, with medium GD severity at the beginning of therapy (according to the number of DSM-5 criteria), the highest level of accumulated debts related to the gambling behavior, the best psychopathological state at baseline (lowest SCL-90R means), the highest mean self-direction and the lowest mean for harm avoidance. Regarding marital status, our results support evidence obtained in other studies that suggests that being married or having a stable partner is related to better therapeutic efficiency (S. Jiménez-Murcia, Fernández-Aranda, et al., 2017; Jiménez-Murcia, Tremblay, et al., 2017). Close relatives and friends of patients with GD usually suffer from the negative effects of the disorder, and they consequently express a positive attitude towards the treatments. These encouraging thoughts should increase the motivation for patients to seek help (Crisp et al., 2004; Dannon et al., 2006), and this better predisposition towards the therapy could also contribute to better adherence by reducing the risk of abandonment and relapse (Gomes and Pascual-Leone, 2015; Tremblay et al., 2015).

Cluster C1 is also characterized by the medium level of DSM-5 symptoms for GD, but by the highest level of accumulated debts from gambling. Financial harm has been described as one of the most commonly reported gambling problems, and gambling-related debt problems have been considered a measure of gambling severity due the strong association between this variable and poor psychosocial functioning (including adverse family impacts, comorbidity with other mental problems and distress). However, studies have also observed a moderator role of sex in the association between gambling severity and cumulate debts among GD patients: (a) among men, as the more severe the negative impacts of the gambling activity, the higher the gambling-related debts; (b) among women, as the financial consequences related with the gambling activity may be less significant for patients with severe/extreme risks of gambling compared to women with a moderate risk of gambling (Håkansson and Widinghoff, 2020). This association could explain why cluster C1 included women with the lowest number of GD symptoms but the highest level of debts. Cluster C1 also presents the best overall psychopathological state. It is known that one primary reason for gambling among women is to alleviate high levels of concurrent symptoms (mainly depression, anxiety and stress) (Marchica et al., 2020). But it is also known that a sub-group of women with better functional mental status show other motivations that explain gambling behavior, such as socialization (Ibáñez et al., 2003; Nuske et al., 2016; Potenza et al., 2006), and this specific group with better baseline functioning should obtain a better benefit from the therapy. Other motivations for gambling behavior in this cluster could also be related to the work situation: C1 grouped the highest prevalence of employed women. Previous studies have observed that active working women with managerial responsibilities could resort to gambling as a way of coping with the specific tensions that their profession causes them, or even as a way of meeting the social demands of their jobs (Toneatto and Wang, 2009). This particular profile could also be highly motivated to receive treatment for their gambling because they want to avoid the negative impacts of gambling problems on their work (such as reduced productivity and results, or damage to their reputation).

Cluster C3 is characterized by poor progression during treatment, with the highest risk of relapse. This cluster includes the highest proportion of women that are unmarried (single or divorced/separated), not actively working, with the highest number of DSM-5 symptoms for GD, the lowest level of accumulated debts due to gambling behavior, the worst psychological state, the highest level of harm avoidance and the lowest self-directedness level. Previous studies have observed that women without a stable partner, in more disadvantaged socioeconomic groups and that are unemployed reported a more severe progression of problematic gambling (Brunborg et al., 2016; R. Granero et al., 2009; Tavares et al., 2001). In this study, the lower level of gambling-related debts in cluster C3 could be related with employment status: being unemployed may explain why these women have a lower capacity to acquire debts. Other studies have also observed that patients with greater socioeconomic difficulties show greater cognitive bias associated with gambling behavior (such as illusion of control, interpretative bias, gambling-related expectancies and distortions of the inability to stop gambling) (Susana Jiménez-Murcia et al., 2020), all of which could be directly associated with greater GD severity and poorer therapeutic efficiency (Ledgerwood et al., 2020; Mallorquí-Bagué et al., 2019).

Regarding psychopathological state, different studies show that a high proportion of women who experience problems with gambling behaviors also have comorbid symptoms (such as anxiety, depression, eating or substance use) (Andronicos et al., 2015; Boughton and Falenchuk, 2007; Dannon et al., 2006; Díez et al., 2014). In fact, GD has been shown to be comorbid with a range of psychosocial and psychopathological symptoms for both genders (Cowlishaw and Kessler, 2016), and it has been reported that more acute psychopathological comorbid states may have serious implications for the treatment benefits (Ledgerwood and Arfken, 2017; Maniaci et al., 2017; Petry et al., 2017): individuals are less likely to terminate the therapy and more likely to relapse if they are suffering from multi-morbidities and harm at baseline. In samples of women with GD, a mediational link has been reported between socioeconomic disadvantage (for example unemployment, low occupational status, low income or poverty), higher difficulties coping with stress, and poorer psychopathological functioning (Boughton and Falenchuk, 2007). This particular profile of women pertaining to low social groups who exhibit distress could use gambling as an means of escape to deal with the effects of their chronic stress [such as physical symptoms (fatigue, eating problems, sleeping disorders or general illnesses), cognitive performance (difficulty concentrating or disorganized thoughts), and other psychological correlates (irritability, low self-esteem, perceived loss of control, feeling helpless, depression or anxiety)] (Blanco et al., 2006; Hodgins and el-Guebaly, 2004; Wenzel and Dahl, 2009). Unfortunately, the progression of the GD among these women leads to even worse comorbid psychopathological symptoms (Susana Jiménez-Murcia et al., 2020; G. Mestre-Bach et al., 2016a, b), and this recursive association (the severity of the gambling-related problems and mental health) could seriously interfere with the efficacy of the therapy (Alvarez-Moya et al., 2011). Ultimately, these women with higher psychological distress may need more time, additional effort, and specific-individualized plans due to the additional care addressed at their comorbid symptoms (Yakovenko et al., 2015).

Regarding personality traits, cluster C3 was characterized by the highest score for harm avoidance and the lowest score for the self-directedness trait. Other studies have identified a profile of women seeking treatment for severe GD with a high dysfunctional psychopathological state, and a personality characterized by lower self-directedness and higher harm avoidance (R. Granero et al., 2009; Roser Granero et al., 2018; Gemma Mestre-Bach et al., 2016a, b). To our knowledge, no empirical study has found any association between this specific profile and poor treatment outcomes in samples of women seeking treatment for GD, but it has been found for males (Roser Granero et al., 2020). Harm avoidance is a personality trait that reflects a tendency towards shyness, passive avoidance behaviors, and concern in anticipation of possible danger, while self-directedness measures responsibility for one's own decisions, the availability of resources to deal with situations, self-esteem and effectiveness. This personality domain is connected with pessimistic and negativistic behavior styles (tendency to be fearful, apprehensive, discouraged and insecure), and its contribution to almost all anxiety disorders has led to the belief that harm avoidance could lead to an anxiety-prone personality (Chen, Lin, Li, Huang, and Lin, 2015; Marco, 2013). High scores for harm avoidance have been shown to contribute to poor treatment efficiency regardless of the type of gambling, the severity of the gambling problem or the duration of the addictive behavior (Maniaci et al., 2017). On the other hand, low scores for self-directedness are related to individuals described as blaming, less responsible, less mature, and driven to react to current circumstances and immediate needs. Among GD samples, low self-directedness appears to be strongly related to high levels of neuroticism-anxiety, more distressed moods, and greater difficulties with the regulation of emotions (Rogier and Velotti, 2018), and this particular profile has significantly contributed to a high risk of relapse or dropout in GD patients (Ramos-Grille et al., 2015). As a whole, these results suggest the need to develop adequate evidence-based therapies that comprise specific strategies aimed at increasing women’s self-esteem and self-efficacy, as well as person-centered techniques to increase women’s self-determination capacities to regulate behaviors to the demands of certain situations in order to avoid relapse.

Cluster C2 included patients with poor progression during treatment, with a high risk of dropout (all the patients in this group dropped out of the CBT, and 19% also had relapses). Regarding sociodemographic features, cluster C2 showed similar features to cluster C3, characterized by a high proportion of unmarried women (without a stable partner) of low social status. Clinically, cluster C2 presented the lowest mean for DSM-5 criteria for GD, medium levels for debts accumulated due to the GD, number of comorbid symptoms measured by the SCL-90R, and in the TCI-R harm avoidance and self-directedness scales. It is important to note that cluster C2 is associated with a high likelihood of dropout, but it is not characterized by the highest mean for the number of DSM-5 criteria or accumulated debts. Previous studies have observed that treatment dropout is related with higher scores for measures reflecting gambling severity (such as impulsivity/addiction, perceived predictive control and gambling-related cognitive distortions) (Fortune and Goodie, 2012; Ledgerwood et al., 2020). Empirical research has also observed that the risk of dropout in patients with GD is related to greater difficulty with self-regulation of behaviors, a high perception of guilt and shame for the addictive behavior, false beliefs about treatment and the presence of emotions of apathy and discouragement (Alvarez-Moya et al., 2011). Our results suggest that it is also possible that women who have not yet reached the most severe levels of affectation by the gambling problem are less aware of the need for therapy, and even consider that they can autonomously control their gambling habit.

Finally, the two treatment outcomes analyzed in this study were the presence of relapses and dropout. There has been much variation in the use of outcome measures of recovery during GD therapy (Pickering et al., 2018). Rates of relapse (and/or abstinence) have been typically reported as the main expected outcomes in a large number of studies, given that no gambling of any nature is defined as the treatment goal. Dropout is also a key therapy outcome, since it has been observed that just over half of individuals who seek treatment for gambling-related problems fail to complete outpatient therapy plans (Ronzitti, Soldini, Smith, Clerici, and Bowden-Jones, 2017). This study contributes with new empirical evidence on women from a person-centered approach, identifying separate empirical clusters that represent differentiated profiles of CBT effectiveness characterized by good progression to recovery versus poor progression to relapse or to dropout.

## Limitations and Strengths

There are some research limitations to this study that could impact the empirical evidence. First, since the data correspond to the outcomes during the CBT, the results could therefore be non-representative over a longer period (there is no way of guaranteeing that abstinence from gambling episodes will persist over time). Second, the data analyzed in the study were recruited from women who attended a specialized care center and fulfilled the inclusion criteria; therefore, our results cannot be generalized to women with gambling-related problems from the general population that do not recur to treatment centers, or men.

Strengths of this study include longitudinal data from a large sample of female GD patients. Another strength is the use of both person-centered and variable-centered approaches, the integration of which has led to a more complete understanding of the processes and patterns of individual profiles.

## Supplementary Information

Below is the link to the electronic supplementary material.

Supplementary file1 (DOCX 13 kb)
