# Supplementary material for: Clustering Treatment Outcomes in Women with Gambling Disorder
Source: J Gambl Stud. 2021 Dec 21;38(4):1469–91. doi: 10.1007/s10899-021-10092-5 (PMC9653370; doi:10.1007/s10899-021-10092-5)
Supplement: Supplementary file 1 — Supplementary file1 (DOCX 13 kb) [file 10899_2021_10092_MOESM1_ESM.docx]

***Table S1 (Supplementary material)***

*Results of the auto-clustering*

| #  Clust. | BIC | ^a^BIC  Change | ^b^Ratio BIC  Changes | AIC | ^a^AIC  Change | ^b^Ratio AIC Changes | ^c^Ratio Distance | Clusters  sample sizes |
| --- | --- | --- | --- | --- | --- | --- | --- | --- |
| 1 | 2788.28 |  |  | 2676.91 |  |  |  | 163 |
| 2 | 2698.29 | -90.00 | 1.000 | 2475.54 | -201.37 | 1.000 | 1.184 | 94/69 |
| 3 | 2650.81 | -47.48 | .528 | 2316.68 | -158.85 | 0.789 | 2.486 | 67/63/33 |
| 4 | 2741.33 | 90.52 | -1.006 | 2295.83 | -20.85 | 0.104 | 1.068 | 67/51/33/12 |
| 5 | 2837.77 | 96.44 | -1.072 | 2280.89 | -14.93 | 0.074 | 1.085 | 51/43/30/27/12 |
| 6 | 2941.03 | 103.26 | -1.147 | 2272.78 | -8.12 | 0.040 | 1.006 | 51/37/27/20/16/12 |
| 7 | 3044.77 | 103.75 | -1.153 | 2265.15 | -7.63 | 0.038 | 1.135 | 37/31/27/20/20/16/12 |
| 8 | 3158.00 | 113.23 | -1.258 | 2267.00 | 1.85 | -0.009 | 1.133 | 31/27/20/20/19/18/16/12 |

*Note.* #Clust: number of clusters. BIC: Schwarz's Bayesian Criterion; AIC: Akaike's Information Criterion.

^a^The changes are from the previous number of clusters in the table.

^b^The ratios of changes are relative to the change for the two cluster solution.

^c^The ratios of distance measures are based on the current number of clusters against the previous number of clusters
